# New Technologies Based on Stem Cell-Therapies in Regenerative Medicine and Reproductive Biology

**DOI:** 10.3390/cells12010095

**Published:** 2022-12-26

**Authors:** Maciej Kurpisz

**Affiliations:** Institute of Human Genetics, Polish Academy of Sciences, Department of Reproductive Biology and Stem Cells, 60-479 Poznan, Poland; maciej.kurpisz@igcz.poznan.pl

Stem cells seem to hold major promise for contemporary medicine, one which could almost be more significant than a discovery of DNA and ultimate its relevance for organismal integration in the past century. Indeed, DNA, its template and its repair may degenerate with age, but stem cells can reproduce infinitely. Although they age together with the body, they retain the promise of immortality. Their renewal has been enhanced through the cell cloning, (in some species) reverting the age of the cells back almost to point “0” [1]. Therefore, we must admit that the first successful mammalian cloning procedure, confirmed by the live birth of Dolly the sheep, has revolutionized both the hierarchy and definition of stemness [2]. In addition, this has also served as the foundation of a new discipline, one which is commonly dubbed regenerative medicine. In more advanced permutations, this is referred to as personalized medicine, owing to the possibilities available for the conversion of adult somatic cells into pluripotential stem cells (iPSc) [3], for which a Nobel Prize has been granted just a few years’ time from that discovery! The theoretical presumption behind such a manipulation has extended the concept that, after reaching pluripotency by adding different sorts of cocktails containing growth and differentiation factors, iPS cells could be re-differentiated in subsequent procedures to the desired fate, supplementing or resettling a pathological organ. Slowly and diligently identifying new next tissue reservoirs step-by-step, it became clear that the whole body (including humans) may contain some sort of stem cells. However, there are some organs that would mostly need supplementation by external cells (we may call this process as “organ rejuvenation”), and to those organs may belong central nervous system (CNS), heart (post-mitotic cardiomyocytes) and pancreas. These organs are also responsible for most disturbing and lifespan limiting civilization diseases, e.g., neurodegenerative diseases (Parkinson disease, Alzheimer’s disease, ALS—amyotrophic lateral sclerosis), diabetes, heart failure and others. Cardiovascular diseases remain the chief contributors to worldwide mortality, but undoubtedly the functioning of the central nervous system has been considered to be the most important feature of *Homo sapiens*, as its impairment may limiting the capacity of human beings to consciously function within family and society. Finally, considering the intensity of civilization, another problem has arisen (perhaps the most complicated one to be corrected by stem cells technology) which is reflected in the global problem of infertility and reproductive health due to the lack of development of germ cells in gonads of both sexes. The mechanisms, however, governing development of germ cells and reproduction are probably less recognized in human species than any other functioning system in a body. 

As we may take a previous concept (organ revitalization through iPSc technology and derived from it re-differentiated cells) seriously, the efficiency of such a procedure (rate conversion of somatic cell into iPSc lines) does not permit us to obtain sufficient amounts of cells to replace major losses incurred during infarction after stroke, or to replace whole organs. Therefore, a diligent search for somatic cell re-programming, little improvements in media contents for long-term in vitro cell culture, and a means of genetic re-programming are still at the top of the Agenda. These topics have been represented in this edition by two papers—Martin-Inaraja et al., 2021 [4], as well as Sowa et al.’s 2021 [5] report. The three other papers are richly directed to problems connected to neurodegenerative disease, either by the way of finding new CNS reservoir candidates [6] or providing insight into possible allogeneic pre-clinical attempts when transplanting cells of CNS out of external sources [7], finally concluding with a review article about the promising ways to influence incurable Alzheimer’s disease by the use of stem cells [8].

Delivery of stem cells to various points of an organism due to rapid progress of endoscopic techniques does not present a large problem, and therefore this was not stressed in any of the articles. Direct administration to CNS may, however, still pose a difficulty. There is more apprehension concerning long-term incorporation of stem cells into a target organ, and again this is far away from an optimal solution and perhaps must be scrutinized in pre-clinical trials. This Special Issue aspired to highlight some aspects of fundamental questions in current stem cells technology.

Stem cell definition of gradual and controlled renewal in principle has changed our view on spermatogonial stem cells, which have become a primary target now for de novo reconstitution of the male gonad with germ cells. Such a situation may happen during non-obstructive azoospermia (NOA), excluding such an individual from reproduction. Spermatogonial stem cells (SSCs) constitute both a self-perpetuating pool in the form of asymmetrical divisions, and also some of them are directed to spermatogenesis which, in a process of proliferation and differentiation (including two points of transition that is from SSCs to spermatocytes and from haploid round spermatids to spermatozoa), provides a haploid gamete. The mitotic process is represented within the spermatogonial pool as the extremely well-developed meiosis proceeds in diploid spermatocytes, which are converted into haploid spermatids. The complexity of the process is enormous and involves approx. 2000 genes, as well as epigenetic phenomena and so-called “imprinting” in which germ cell progeny acquires a new “genetic stamp”, erasing the old one. It is difficult to say therefore that we control this process—in many of cases there are difficulties in controlling the in vitro environment for a sufficient number of essential factors providing supplies through approx. 76 days, from the first step to the final accomplishment of spermatogenesis into spermatozoa. As epigenetic mechanisms act in parallel, influencing gamete imprinting for which each sex governs each set of genes in reproductive development, it becomes clear that, even by obtaining finally differentiated spermatozoa, there is no guarantee that they may provide live offspring. On the contrary, reconstituted in situ spermatozoa, even when applied to the seminiferous microenvironment of another donor, may result in an aberrant development/physiology of offspring. Despite these facts, successful in vitro derivation and propagation of spermatogonial stem cells from mouse pluripotent stem cells has been obtained [9]. In a mouse model, male germ cells development has been described into three phases. The first one begins with primordial stem cells (PGCs), which could be paralleled with the fetal germ cells (FGCs) described in the paper Martin-Inaraja et al. [4] published in our issue. PGCs may then differentiate into gonocytes with epigenetic reprogramming. The second phase involves the gonocyte-to-pro-spermatogonium stage, eventually concluding in SSCs differentiation which may culminate by the third phase of SSCs initiated spermatogenesis. There is no surprise that the fundamental question of the early stage of male germ cells development/differentiation, specifically in humans, may require a high level of attention. Specifically, in vitro culture of early stages germ cells is highly demanding and initially xenogeneic feeder cells were essential. As a number of growth/transcription factors, hormones, paracrine factors etc. was gradually identified, new generation culture media together with semi-solid coated surfaces have been applied. In the paper of Martin-Inaraja et al. [4], authors investigated the influence of different compositions of media, the so-called Zhou vs Shinohara medium together with a range of coating solid substrates (laminin, gelatin, vitronectin, matrigel). Human fetal tissues were allowed to be used from elective abortions (early stage of pregnancy), offering a unique material for the undertaken experiments which allowed us to produce clear-cut results (SSCs could be further differentiated to spermatid-like cells) in a feeder-free culture system.

The authors rightly underline that media compositions tested in the study would allow the sustained male hFGCs long-term culture to not change its identity, something which failed in cases of previous studies. Although their results may provide imminent help in in vitro cultures of gametogenesis, we should further maintain that human primates’ completion of spermatogenesis may require a “native” seminiferous tubules microenvironment to provide fully functional spermatozoa. The efficiency of such a procedure may be very low as, using human SSCs transplanted to *Rhesus macaques* [10], their ejaculated sperm with donor paternal origin impregnated 7 out of 81 obtained embryos (that is approx. 8%). Human male fetal germ cells will be for a long time a model for human gametogenesis in vitro, and as such the maintenance of their identity is critical to undertake these studies in a reliable way. 

Classical conversion of somatic cells into inducible pluripotent stem cells includes overexpression of the four reprogramming factors (OCT-4, SOX-2, KLF-4 and c-MYC). First-generation technologies were based on retroviral and lentiviral systems allowing for highly efficient reprogramming, but lacked the necessary controls over host–genome activation. Second-generation technologies used non-integrating episomal DNA plasmids which were transgene-free but lacked the high reprogramming efficiency of viral vectors. Third generation technologies used non-integrating RNA viruses which have been widely used until now, termed Sendai viruses technology. These RNA viruses produce integration-free iPSCs with a high reprogramming rate. The next era began with mimicking the effects of the selected genes overexpression (specifically *OCT*-4) by small molecule compounds. Small molecules may inhibit or activate the function of proteins, which can be often reversible and controlled through a well-tuned small molecules cocktail (permeable to the cell). To identify small molecules facilitating reprogramming, a system in *Oct-4* promoter-driven GFP expression in mouse embryonic fibroblasts was used. After screening approx. 10,000 small molecules, we identified: Forskolin (FSK), 2-Methyl-5-hydroxytryptamine (2-Me-5HT) and D4476 that could have substituted *Oct-4* action [11]. Previously, the same group identified a small molecule combination called as VC6T (VPA—valproic acid; CHIR—CHIR99021, 616452, tranylcypromine) which enabled reprogramming with a single gen, *Oct4*. As usual, it took a long time to manage chemical reprogramming of adult somatic cells into pluripotent stem cells in humans, but finally the process succeeded using small molecules, including epigenetic regulators. Screened small molecules included those universally assigned previously in the mouse system—valproic acid, tranylcypromine and cell signaling inhibitors CHIR99021 and 616452. In such cases, human chemically induced pluripotent stem cells (hCiPS) were generated from human adult adipose-derived mesenchymal stromal cells (hADSCs) and human adult skin dermal fibroblasts (hASFs) [12]. Finally, the intention of personalized reprogramming somatic cells into iPS and then their re-differentiation to the desired phenotype has been replaced by direct reprogramming, omitting a stage of pluripotency. An example of this was a reprogramming of human umbilical cord mesenchymal stem cells (hUCMSC) into induced neurons (iNs). This was achieved by the expression of several transcription factors, such as sex-determining region Y-box w (*Sox-2*), achaete-scute homolog-1 (*Ascl1*) and neurogenin 2 (*Neurog2*). This combination was sufficient however, for up to turn 50% of transfected hUCMSC into iNs with multiple overlapping neuronal phenotypes and limited functionality in electrical properties [13]. Therefore, a direct re-programming may alter some important epigenetic balances that cannot not totally erased and which are de novo difficult to re-establish after such manipulation.

Nevertheless, both above-described schemes have been explored by the authors of our issue. In the first example [5], they have screened more than 10,000 chemical molecules for the ability to induce lipid accumulation in human dermal fibroblasts (HDFs). A molecule, STK287794 has been defined for this purpose, where human fibroblasts were converted into adipocytes; these were so-called chemically compound converted adipocytes (CCCAs), which were then screened for secretion of adiponectin and leptin. As their functional characteristics seem to be promising, it should be noteworthy to study only obtained reprogrammed adipocytes for regeneration therapy in reconstructing soft tissue defects by fully compatible tissue.

A second paper represents the application of direct re-differentiation of hair follicle-associated (HAP) cells located in the bulge area of hair follicles [6] to neurons by dopaminergic-neuron-maturation medium (1 and 2). Though it does not seem to require much effort in the case of HAP cells to observe in maturation media secretion of dopamine and the expression of tyrosine hydroxylase, it seems evident that these cells should be used at least in pre-clinical studies to ameliorate Parkinson disease as it was previously shown in case of sciatic nerve regeneration and/or spinal cord injured rats (improvement of locomotor function) through HAP implantation [14]. 

The most interesting part of this Special Issue has been dedicated to novel treatment of neurodegenerative diseases. These types of disorders constitute the most difficult tasks for contemporary cellular therapies since they may affect the central nervous system. However, the majority of cases of neurodegenerative diseases are not treatable in a standard way. Amyotrophic lateral sclerosis (ALS) is rare but one of the deadliest diseases, with the lifetime risk 1:350. However, global incidence states its frequency for 1.68 per 100,000 person-years, varying according to the region. There is familial ALS, which constitutes approx. 10–15% of cases, and among them 70% helped to detect mutated genes involved in this pathology. There have been more than 40 ALS genes discovered, among which most common and penetrant mutations are *C9orf72*, *TARDP*, *SOD1* and *FUS* genes [15]. Three of them impair RNA metabolism, while mitochondrial dysfunction has been triggered by *SOD1* mutation and is a central characteristic for ALS increased oxidative stress, in consequence developing autoimmune reactions concerning anterior horns of spinal cord. Progressive dysfunction of the motor neurons leads to skeletal muscle weakness and atrophy. The late phase of the disease ends in a failure of intercostal muscles and respiratory deficiency and death. Transplantation of several types of stem cells for treatment of ALS has led in numerous Phase I/II clinical trials to quite inconclusive results, and we identified 670 references and 90 full-text studies [16]. Patients were treated, receiving mesenchymal, neural or mononuclear cells of bone marrow origin but not glial cells, which were used in our pre-clinical study published in this Special Issue [7]. The route of administration seems to be also of a great importance when considering sophisticated CNS environments. Although published meta-analyses encompassed different administration routes such as intrathecal, intrathecal+intramsucular, intravascular or intraspinal (in our case it was *cisterna magna* administration), the interventions provided only transient positive effects on clinical progression while worsening respiratory function. In our case of pre-clinical scenario with glial cells, they were accompanied by immunosuppressive/immunomodulatory agents. It occurred, however, that semiallogeneic transplantations were the best surviving implants provoking a speculation that, while administering the cells from other individuals (fetal cells), HLA restrictions must be taken into account. The next paper in our issue was an attempt to summarize overall stem cell therapy in Alzheimer’s disease [8]. Unlike in SLA, the authors are optimistic concerning treatment of Alzheimer’s disease (AD). In their view (review paper), stem cell therapy improves memory loss and cognitive deficits in animal models. There is a prevailing view that stem cells may stimulate neurogenesis and inhibit apoptosis through the regulation of complex systems of autocrine and paracrine cytokines. It has been also reflected in our earlier clinical experiments, in which Duchenne dystrophy children were treated with combination of mesenchymal and myogenic cells, that cellular therapy significantly lowered cytokine levels and induced regenerative waves in EMG of treated muscles [17]. This phenomenon would argue for the universal pro-inflammatory nature of musculo-nervous diseases reflected e.g., in amyloid beta plaques cumulation within the CNS. Oxidative stress, immune-related reactions, inflammation and apoptosis seem to be inadvertently linked, perpetuating neurodegenerative diseases. It is further documented (pre-clinical animal studies) that stem cell therapy may stimulate the reconstruction of synaptic connections through microglial activity and remyelination. In such a scenario, glial cells may find a prominent place in reconstructive therapy. It has to be under consideration, however, that animal pre-clinical studies may not be fully adequate and extrapolated to humans. Specifically, long-term maturation of CNS in humans, longer in any other known species which is connected to a high degree of specialization and the development of the neocortex, may be a severe challenge to the CNS environment when humans comparing to other mammals. On the other side of the coin, it has to be emphasized that microbial infections staying behind Alzheimer’s diseases seem to be pretty universal, as HSV-1 of DNA has been identified within amyloid plaques while *Borrellia* seem to be responsible for induction of Lyme disease and dementia [8]. Therefore, we are convinced that our Special Issue may enlighten issues stem cell application to the readers and will be met with great interest by the public.
